# Stress Granule Assembly in Pulmonary Arterial Hypertension

**DOI:** 10.3390/cells13211796

**Published:** 2024-10-30

**Authors:** Kosmas Kosmas, Aimilia Eirini Papathanasiou, Fotios Spyropoulos, Rakhshinda Rehman, Ashley Anne Cunha, Laura E. Fredenburgh, Mark A. Perrella, Helen Christou

**Affiliations:** 1 Department of Pediatrics, Division of Newborn Medicine, Brigham and Women’s Hospital and Harvard Medical School, Boston, MA 02115, USA; 2 Department of Pathology, Beth Israel Deaconess Medical Center and Harvard Medical School, Boston, MA 02115, USA; 3 Regeneron Pharmaceuticals, Greenburgh, NY 10591, USA; 4 Department of Medicine, Division of Pulmonary and Critical Care, Brigham and Women’s Hospital and Harvard Medical School, Boston, MA 02115, USA

**Keywords:** PAH, stress granules, G3BP1, Caprin1, ACTZ, ISRIB, vascular smooth muscle cells

## Abstract

The role of stress granules (SGs) in pulmonary arterial hypertension (PAH) is unknown. We hypothesized that SG formation contributes to abnormal vascular phenotypes, and cardiac and skeletal muscle dysfunction in PAH. Using the rat Sugen/hypoxia (SU/Hx) model of PAH, we demonstrate the formation of SG puncta and increased expression of SG proteins compared to control animals in lungs, right ventricles, and soleus muscles. Acetazolamide (ACTZ) treatment ameliorated the disease and reduced SG formation in all of these tissues. Primary pulmonary artery smooth muscle cells (PASMCs) from diseased animals had increased SG protein expression and SG number after acute oxidative stress and this was ameliorated by ACTZ. Pharmacologic inhibition of SG formation or genetic ablation of the SG assembly protein (G3BP1) altered the SU/Hx-PASMC phenotype by decreasing proliferation, increasing apoptosis and modulating synthetic and contractile marker expression. In human PAH lungs, we found increased SG puncta in pulmonary arteries compared to control lungs and in human PAH-PASMCs we found increased SGs after acute oxidative stress compared to healthy PASMCs. Genetic ablation of G3BP1 in human PAH-PASMCs resulted in a phenotypic switch to a less synthetic and more contractile phenotype. We conclude that increased SG formation in PASMCs and other tissues may contribute to PAH pathogenesis.

## 1. Introduction

Pulmonary arterial hypertension (PAH) is a progressively debilitating and often fatal disease characterized by increased pulmonary vascular resistance, and right ventricular hypertrophy (RVH) and failure [1]. Specifically in pulmonary artery smooth muscle cells (PASMCs), the PAH phenotype is characterized by increased proliferation and migration, apoptosis resistance, and loss of differentiation (dedifferentiation), as seen by significantly decreased contractile and increased proliferative and synthetic markers [2,3]. Stress granules (SGs) are large, non-membranous cytoplasmic entities that occur in response to environmental stress, including hypoxia, viral infection, oxidative stress, and heat. SGs contain mRNAs, RNA-binding proteins (RBPs), and translation initiation complexes, and regulate gene expression by delaying the translation of specific transcripts via phosphorylation of the translation initiation factor eIf2α at serine 51 [4,5,6]. SG components include G3BPs (Ras-GAP SH3-domain-binding protein-SG assembly factors [7,8,9], and Caprin1 (Cell cycle associated protein 1), a core SG component that binds G3BP1 and promotes SG formation [10]. Abnormal SG development and mutations in SG genes are associated with many diseases, and pharmacologic targeting of SG-dependent pathways is currently being explored as a potential therapeutic opportunity in cancer [11,12,13,14] and neurodegenerative disorders [15,16], but their role in PAH pathogenesis is not known. The well-established Sugen 5416/ hypoxia adult rat model of severe PAH (SU/Hx-PH) recapitulates several features of human PAH, including PASMC phenotypic switch, RV failure, and skeletal muscle dysfunction, and treatment with acetazolamide (ACTZ) ameliorates the disease [3,17,18]. In this clinically relevant rodent model and in samples from PAH patients we evaluated the formation of SG component puncta in vitro and in vivo and studied the effect of ACTZ and genetic ablation of the SG assembly protein (G3BP1). Our findings support a potential link between increased stress granule assembly and PAH pathogenesis.

## 2. Materials and Methods

### 2.1. Human IPAH and Control Samples

Samples of human lungs were obtained through the Pulmonary Hypertension Breakthrough Initiative (PHBI) (http://www.ipahresearch.org accessed on 15 June 2023).

A standardized tissue processing protocol was previously described [19]. Patient characteristics are provided in Supplementary Table S1. All samples were de-identified, coded, and matched for age and gender as closely as possible.

### 2.2. Animals and Experimental Models of Pulmonary Hypertension

Adult (12-week-old) male Sprague–Dawley rats (Charles River Laboratories) were acclimatized for 2–3 days prior to the experiments. PH was induced as previously described [3,20]. Animals were given a subcutaneous injection of 20 mg/kg Sugen 5416 in dimethyl sulfoxide (DMSO), placed in hypoxic condition (9% O_2_) for 3 weeks, and then returned to normoxia. An OxyCycler controller (BioSpherix, Redfield, NY, USA) was used to control oxygen at a level of 9% ± 0.2%, and ventilation was adjusted with a fan and port holes to remove CO_2_ and ammonia. Control animals were injected with an equal volume of vehicle (DMSO) in normoxic condition. The endpoints of the study were 24 days after Sugen injection, as depicted in Supplementary Figure S1. All experimental procedures were conducted in accordance with the guidelines of the American Physiologic Society and the National Institutes of Health and were approved by the Harvard Institutional Animal Care and Use Committee and the Harvard Medical Area Standard Committee on Animals (Brigham and Women’s Institutional Animal Care and Use Committee).

### 2.3. Treatment Protocol

Experimental groups: Ctrl (normoxia control with vehicle injection), SU/Hx (Sugen/Hypoxia), SU/Hx + ACTZ (Sugen/Hypoxia treated with ACTZ; Supplementary Figure S1). Rats were randomized into control and ACTZ treatment groups. ACTZ (1.7 mg/mL) was added to the drinking water. Sucrose (5% *w*/*v*) was added to treated and control animals to increase water intake. Water consumption was monitored and estimated to be ~20 mL per rat per diem. The treatment protocol consisted of administration of ACTZ from days 7 to 24 [3,17].

### 2.4. Hemodynamic Measurements

Hemodynamic measurements were performed as previously described [20,21,22]. Animals were anesthetized with 3% isoflurane inhalation, a tracheostomy was performed, and rats remained mechanically ventilated on a rodent ventilator (Harvard Apparatus, tidal volume 1 mL/100 g body weight, 60 breaths/min). A small incision was made in the abdominal wall and the diaphragm was exposed. A 23-gauge heparinized butterfly needle with tubing connected to a pressure transducer was inserted initially into the right and then into the left ventricle, and pressure measurements were obtained and recorded with PowerLab monitoring hardware and software (ADInstruments, Colorado Springs, CO, USA). Mean right ventricular systolic pressure (RVSP) and left ventricular systolic pressure (LVSP) (in mmHg) over the first 10 stable heartbeats were recorded. Right ventricular hypertrophy (RVH) was assessed by weighing RV mass and expressed as Fulton’s Index (FI, ratio of right ventricular weight to the left ventricular + septal weight) or as the ratio of right ventricular weight to total body weight (RV/BW).

### 2.5. Rat Tissue Isolation

After hemodynamic measurements were completed, the rats were sacrificed by exsanguination. Lungs, right and left ventricles, and soleus muscles were harvested immediately. Samples were snap-frozen and stored at −80 °C until processing.

### 2.6. Immunohistochemistry

Lungs, right and left ventricles, and soleus muscles were fixed in 4% paraformaldehyde before paraffin embedding. Paraffin sections of 5 µm thickness were deparaffinized in 2 changes of xylene and rehydrated through a graded ethanol series. This was followed by antigen retrieval and antibody incubation. Sections were mounted, followed by confocal microscopy.

### 2.7. Cell Lines and Culture Conditions

Human Pulmonary Arterial Smooth Muscle Cells (HPASMCs) were cultured in complete SmBM Lonza media supplemented with antibiotic/antimycotic solution 1:100 and Rat Pulmonary Arterial Smooth Muscle Cells (RPASMCs) were cultured in high-glucose DMEM supplemented with 10% fetal bovine serum (FBS), 100 IU/mL penicillin, and 100 µg/mL streptomycin and were grown at 37 °C in 95% humidified air with 5% CO_2_ to 70% confluency prior to experimentation. Both cell lines checked negative for Mycoplasma using the MycoAlert Mycoplasma Detection Kit (Lonza), according to the manufacturer’s instructions.

### 2.8. Western Blot Analysis

Tissues and cells were homogenized and lysed using RIPA buffer (Cell Signaling Technology, Danvers, MA, USA) containing phosphatase and protease inhibitors. Equal amounts of total protein were resolved on polyacrylamide SDS gels and transferred to polyvinylidene difluoride membranes. Blots were blocked in Tris Buffered Saline with Tween-20 (TBST) with 5% *w*/*v* nonfat dry milk and then subjected to immunolabeling with primary antibodies overnight at 4 °C. The membranes were washed and then incubated with horseradish peroxidase-conjugated secondary antibodies at room temperature for 1 h. Bands were detected using SuperSignal West Pico Plus chemiluminescence substrate (Thermo Fisher Scientific, Waltham, MA, USA), photographed with Biorad ChemiDoc Imaging System and quantified using ImageJ (Version 1.53k) analysis software.

### 2.9. WST-1 Proliferation Assay

HPASMCs and RPASMCs were seeded on a 96 well plate, starved for 24 h (0.5% FBS), and then treated with 20% FBS in fresh medium. The cell number in each well at 48 or 72 h was counted using the WST-1 kit (Roche, Basel, Switzerland) according to the manufacturer’s instructions. Supernatant was removed, and then cells were incubated with 100 µL of the fresh medium along with 10 µL of the WST-1 reagent, for 2 h in dark at 37 °C. A microplate reader was used to determine the optical density (OD) at 450 nm.

### 2.10. siRNA Transfections

Cells were transfected with Lipofectamine-RNAiMAX Reagent (Invitrogen, Waltham, MA, USA). siRNA Smart pools targeting human G3BP1 (10146) and rat G3BP1 (171092), and non-targeting controls (Dharmacon, Lafayette, CO, USA) were used at 25 nM final concentration.

### 2.11. Antibodies and Reagents

Antibodies used: G3BP1 (rabbit; Proteintech, Rosemont, IL, USA), G3BP1 (mouse; Abcam), Caprin1 (rabbit; Proteintech), Phospho-eIF2a(S51) (rabbit; Cell Signaling, Danvers, MA, USA), Connexin 43 (mouse; Millipore Sigma, Burlington, MA, USA), Desmin (rabbit; Cell Signaling), α Smooth muscle actin (mouse; Millipore), PARP (rabbit; Cell Signaling), Caspase 3 (rabbit; Cell Signaling), vinculin (rabbit; Cell Signaling), GAPDH (rabbit; Cell Signaling). Additional reagents included: Sugen 5416 (Millipore Sigma), ACTZ (Spectrum, Gardena, CA, USA), NaAsO_2_ (sodium arsenite) (Millipore Sigma), and DMSO (Millipore, Sigma).

### 2.12. Confocal Microscopy

Cells were seeded on 4-chamber tissue culture glass slides overnight, fixed with 4% paraformaldehyde, then incubated with primary antibodies overnight at 4 °C, washed, and incubated with fluorophore-conjugated secondary antibodies for another hour. Tissues were processed for immunofluorescence analysis and then incubated with primary antibodies overnight at 4 °C, washed, and incubated with fluorophore-conjugated secondary antibodies for another hour. Nuclei were visualized with DAPI (40,6-diamidino-2-phenyl-indole) staining. Images were captured with a FluoView FV-10i Olympus Laser Point Scanning Confocal Microscope using a 60X objective lens. Confocal filters (Excitation/Emission nm) used for microscopy imaging were: 490/525 (Alexa-Fluor488), 578/603 (Alexa-FluorA568), 590/617 (Alexa-Fluor594) (Invitrogen), 358/461 (DAPI). Image analysis was performed using CellProfiler (cellprofiler.org), an open source software for image analysis [23].

### 2.13. Statistical Analysis

Statistical analyses were performed with GraphPad Prism version 10.0.2 (GraphPad Software). Normally distributed data were analyzed for statistical significance with Student’s unpaired t-test and multiple comparisons were made with one-way and two-way ANOVAs with Bonferroni correction. Data are presented as mean and standard error of the mean (SEM). Statistical significance was defined as *p* < 0.05.

## 3. Results

### 3.1. SG Puncta and Upregulation of SG Components in Lungs from Animals with SU/Hx-Induced PH and Decrease in SG Components After ACTZ Treatment

**Figure 1:**
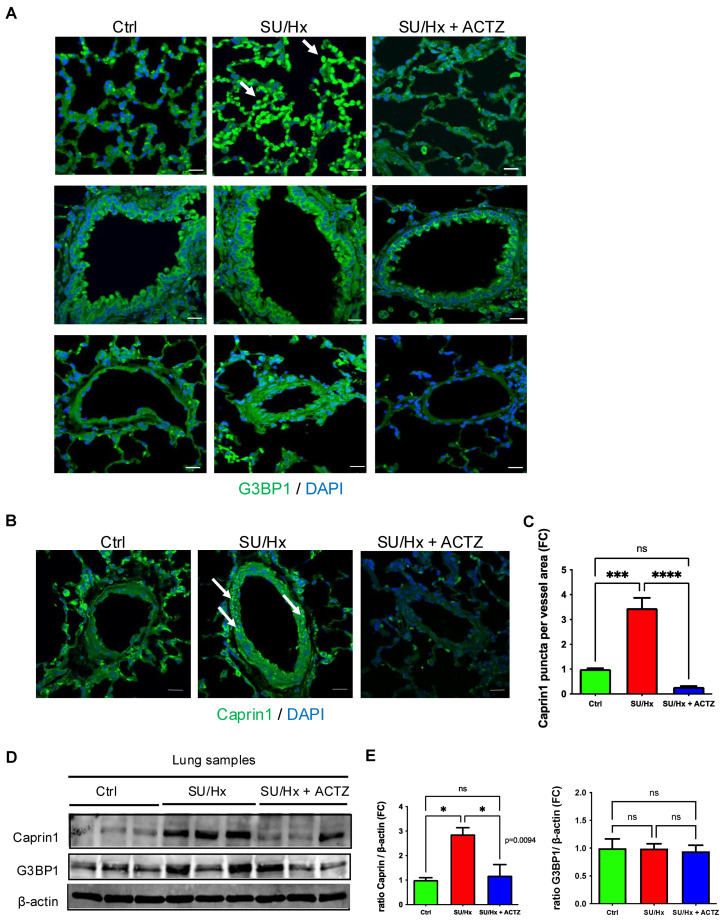
SG puncta in lungs from animals with SU/Hx-induced PH compared to controls and with ACTZ treatment. Paraffin-embedded sections of lungs stained with G3BP1. (**A**) G3BP1 puncta in pulmonary alveoli (**upper** panels) indicated by arrows; higher G3BP1 expression in airways (middle panels) and in pulmonary blood vessels (**lower** panels) in lungs from animals with SU/Hx-induced PH compared to controls and those with ACTZ treatment. Paraffin-embedded sections of lungs stained with Caprin1 (**B**) antibodies. Arrows indicate Caprin1 puncta, quantified with CellProfiler (**C**)**.** Nuclei were visualized with DAPI staining. Scale bar, 20 μm. (**D**) Immunoblots showing upregulation of Caprin1 and no difference in G3BP1 expression levels in lung lysates of SU/Hx animals compared to SU/Hx-ACTZ-treated and controls, and quantitative analysis of immunoblots. (**E**) β-actin was used as a loading control, *n* = 3 animals per group; ns: not significant, * *p* < 0.05, *** *p* < 0.001, **** *p* < 0.0001.

We subjected adult rats to a single subcutaneous injection of Sugen 5416 followed by exposure to hypoxia for three weeks to induce PH and a subset of animals were treated with ACTZ (Supplementary Figures S1 and S2). As we previously reported, SU/Hx animals had significantly increased RVSP, Fulton’s Index (FI) and right ventricular (RV) weight to body weight (BW) ratios (RV/BW) compared to controls and ACTZ-treated SU/Hx animals (Supplementary Figure S2). Immunofluorescence analysis showed the formation of G3BP1 puncta in pulmonary parenchyma and increased G3BP1 abundance in airways and pulmonary blood vessels in lungs from animals with SU/Hx-induced PH compared to controls (Figure 1A). Also, Caprin1 staining revealed an increased number of Caprin1 puncta in pulmonary blood vessels compared to controls (Figure 1B,C). Interestingly, in vivo treatment with ACTZ decreased the formation of G3BP1 puncta in pulmonary parenchyma, G3BP1 abundance, and Caprin1 puncta in pulmonary vessels of SU/Hx animals (Figure 1A–C). In addition, there was higher abundance of Caprin1 by Western analysis in lung lysates from SU/Hx animals compared to SU/Hx treated with ACTZ and controls, but no difference in G3BP1 protein levels (Figure 1D,E).

### 3.2. Ribonucleoprotein (RNP) Granulopathy in Right Ventricles from Animals with SU/Hx-Induced PH Compared to ACTZ Treatment

**Figure 2:**
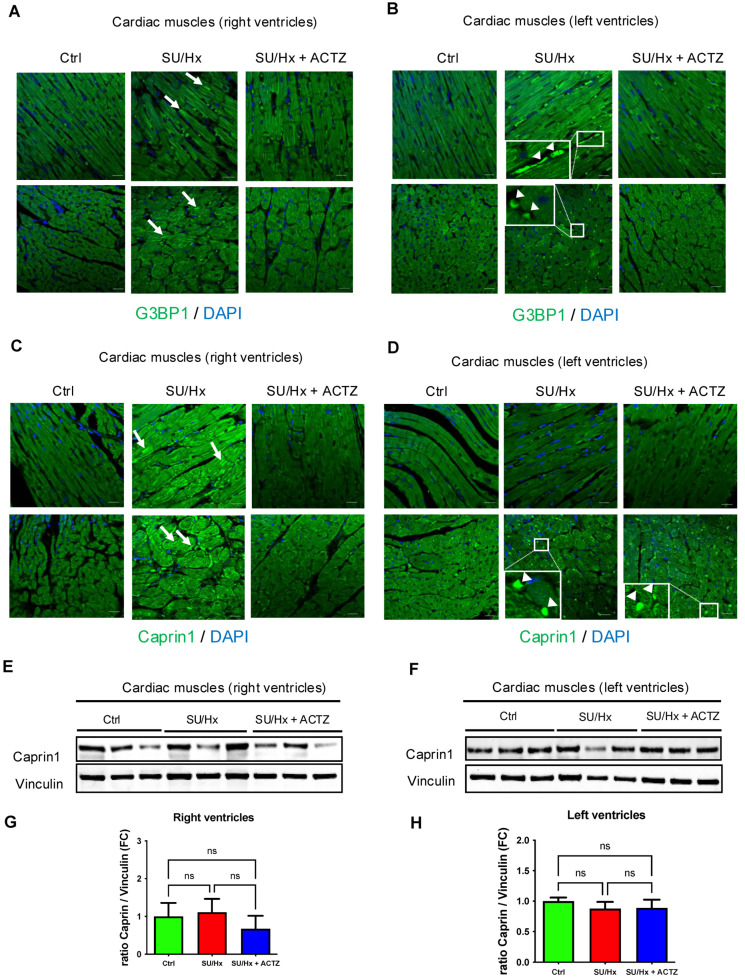
Sarcoplasmic G3BP1 and Caprin1 puncta in right ventricles from animals with SU/Hx-induced PH compared to controls and with ACTZ treatment. Paraffin-embedded sections of right ventricles (longitudinal upper panels and transverse lower panels) (**A**); left ventricles (longitudinal upper panels and transverse lower panels) (**B**) stained with G3BP1 antibodies; right ventricles (longitudinal upper panels and transverse lower panels) (**C**); and left ventricles (longitudinal upper panels and transverse lower panels) (**D**) stained with Caprin1 antibodies. Arrows indicate G3BP1 and Caprin1 puncta respectively. Arrowheads indicate debris at the periphery of the cardiac muscle fibers. Nuclei were visualized with DAPI staining. Scale bar, 20 μm. (**E**) Immunoblots showing Caprin1 protein expression in right ventricles lysates of SU/Hx animals compared to SU/Hx-ACTZ treated and controls, and quantitative analysis of immunoblots (**G**)**.** (**F**) Immunoblots showing Caprin1 protein expression in left ventricles lysates of SU/Hx animals compared to SU/Hx-ACTZ treated and controls, and quantitative analysis of immunoblots (**H**)**.** Vinculin was used as a loading control, *n* = 6 animals per group; ns: not significant.

Staining with the SG markers G3BP1 and Caprin1 of paraffin-embedded right and left ventricle tissue sections from SU/Hx, controls, and SU/Hx + ACTZ animals revealed the following findings: G3BP1 and Caprin1 puncta were more apparent in the sarcoplasm in right ventricle sections from SU/Hx animals (Figure 2A,C upper panels, arrows) and in the subsarcolemmal region (Figure 2A,C lower panels, arrows) demonstrating RNP granulopathy compared to right ventricles from controls and ACTZ-treated animals. In contrast, in tissue sections from SU/Hx animals’ left ventricles there were no G3BP1 and Caprin1 puncta (Figure 2B,D) and no difference in Caprin1 protein levels (Figure 2F,H) compared to controls. Western analysis did not reveal differential abundance of Caprin1 in right-ventricle lysates from SU/Hx-induced PH animals compared to controls or ACTZ-treated animals (Figure 2E,G) because the formation of SG puncta depends on the distribution and abundance of Caprin1 in these puncta, not the total protein levels of Caprin1 in these tissues.

### 3.3. RNP Granulopathy and Increased SG Markers in Soleus Muscles from Animals with SU/Hx-Induced PH and Decrease After ACTZ Treatment

**Figure 3:**
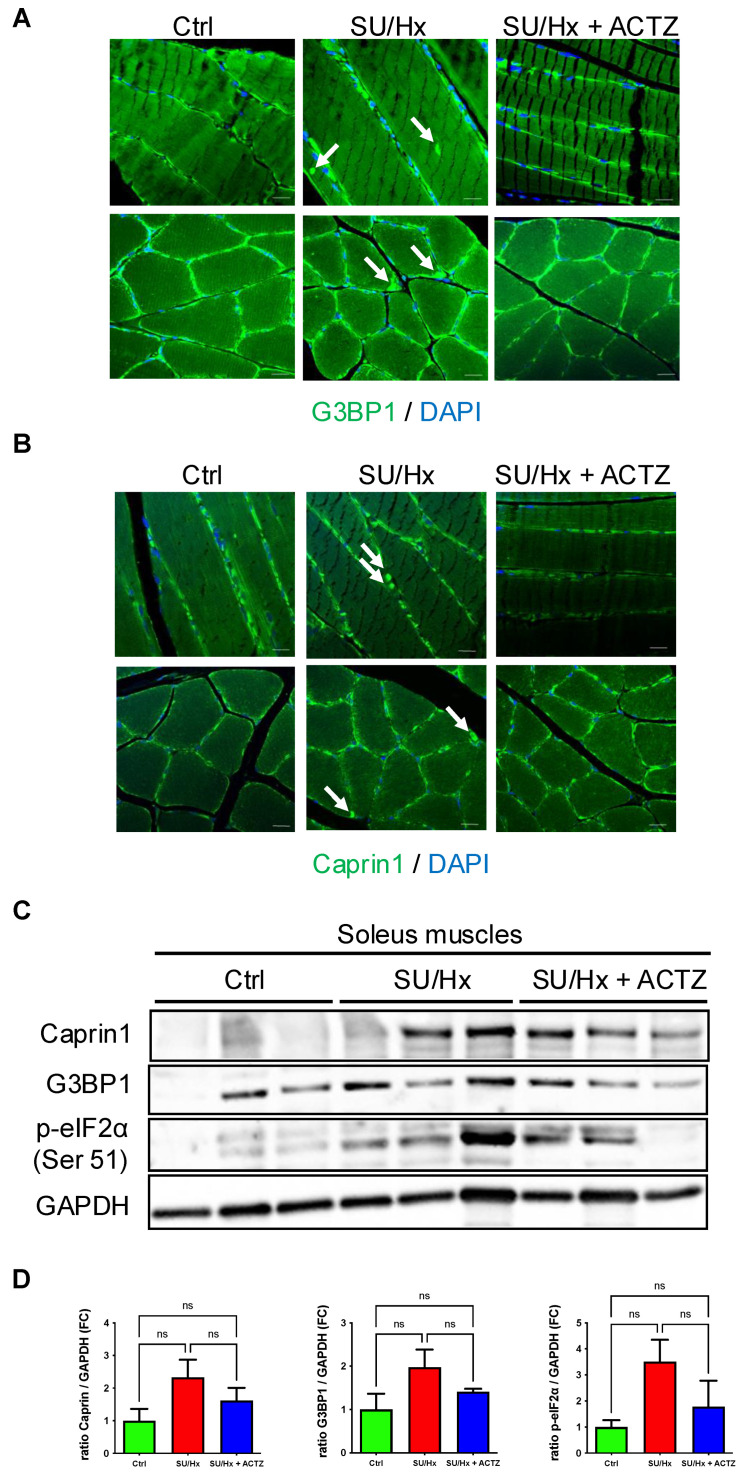
Sarcoplasmic G3BP1 and Caprin1 puncta in soleus muscles from animals with SU/Hx-induced PH compared to controls and with ACTZ treatment. Paraffin-embedded sections of soleus (longitudinal upper panels and transverse lower panels) stained with G3BP1 (**A**) and Caprin1 (**B**) antibodies. Arrows indicate G3BP1 and Caprin1 puncta, respectively. Nuclei were visualized with DAPI staining. Scale bar, 20 μm. (**C**) Immunoblots showing upregulation of SG components’ (Caprin1, G3BP1, p-eIF2α) protein expression in soleus lysates of SU/Hx animals compared to SU/Hx-ACTZ-treated animals and controls, and quantitative analysis of immunoblots (**D**)**.** GAPDH was used as a loading control; *n* = 6 animals per group; ns: not significant.

We next stained paraffin-embedded soleus muscles from SU/Hx animals, controls, and ACTZ-treated animals with G3BP1 and Caprin1 (Figure 3A,B). We found that in skeletal muscle fibers from SU/Hx soleus muscle sections, G3BP1 and Caprin1 puncta became apparent in the sarcoplasm (Figure 3A,B upper panels, arrows) and in the subsarcolemmal region (Figure 3A,B lower panels, arrows) demonstrating RNP granulopathy compared to controls and ACTZ-treated animals. 

We also found a trend of SG components being increased (G3BP1 and Caprin1) by Western analysis in soleus muscle tissue lysates from animals with SU/Hx-induced PH, compared to controls and ACTZ-treated animals. In addition, there was higher abundance of phospho-eIF2a (regulator of SG formation [6]) in SU/Hx-derived tissues and in vivo treatment with ACTZ led to a decrease (Figure 3C,D).

### 3.4. Increased Number of SGs in PASMCs from Rats with SU/Hx-Induced PH and Decreased Number of SGs After ACTZ Treatment

**Figure 4:**
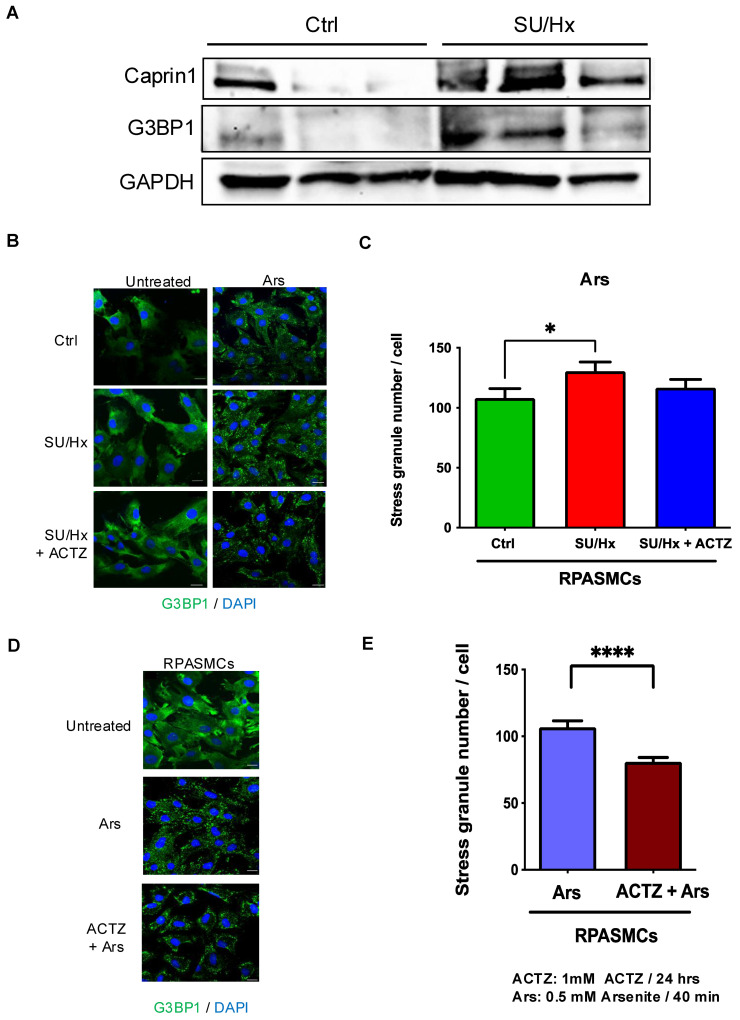
RPASMCs from SU/Hx animals have increased SG markers at baseline and more SGs after acute oxidative stress, and in vivo and in vitro treatment with ACTZ results in lower numbers of SGs. (**A**) Immunoblot showing upregulation of SG components (Caprin1 and G3BP1) in RPASMC lysates isolated from SU/Hx animals compared to controls. GAPDH was used as a loading control. (**B**) RPASMCs from SU/Hx animals, SU/Hx animals treated with ACTZ, and controls were treated with 0.5 mM arsenite for 40 min and stained for G3BP1 to detect SGs, quantified with CellProfiler. (**C**) (*n* = 55 cells/cell type). (**D**) RPASMCs were pre-treated with 1 mM ACTZ for 24 h, then treated with 0.5 mM arsenite for 40 min and stained for G3BP1 to detect SGs, quantified with CellProfiler (**E**) (*n* = 125 cells/cell type). Nuclei were visualized with DAPI staining. Scale bar, 20 μm; **** *p* < 0.0001. * *p* < 0.05.

We isolated RPASMCs from animals with SU/Hx-induced PH, and by Western analysis we found a higher abundance of multiple SG components including G3BP1 and Caprin1 in SU/Hx-derived RPASMCs at baseline compared to controls (Figure 4A). The same cells were exposed to oxidative stress (treatment with arsenite (NaAsO_2_)) and SG assembly was evaluated by G3BP1 staining. As shown in Figure 4B,C, RPASMCs from SU/Hx animals have 25% more SGs after acute oxidative stress compared to cells isolated from control rats. Similarly, in vivo treatment with ACTZ led to a reduced number of SGs in response to arsenite treatment in RPASMCs from SU/Hx animals (Figure 4B,C). We then evaluated whether ACTZ treatment in vitro would inhibit SG formation induced by arsenite in primary RPASMCs. Arsenite treatment strongly induced formation of G3BP1-positive SGs in normal RPASMCs, and pre-treatment with ACTZ significantly reduced the amount of SG formation per cell by 20% (Figure 4D,E). These results support that ACTZ inhibits SG formation in response to oxidative stress in RPASMCs.

### 3.5. ISRIB Treatment Results in Lower Number of SGs After Oxidative Stress and Restores the Contractile Phenotype of RPASMCs from SU/Hx Animals

**Figure 5:**
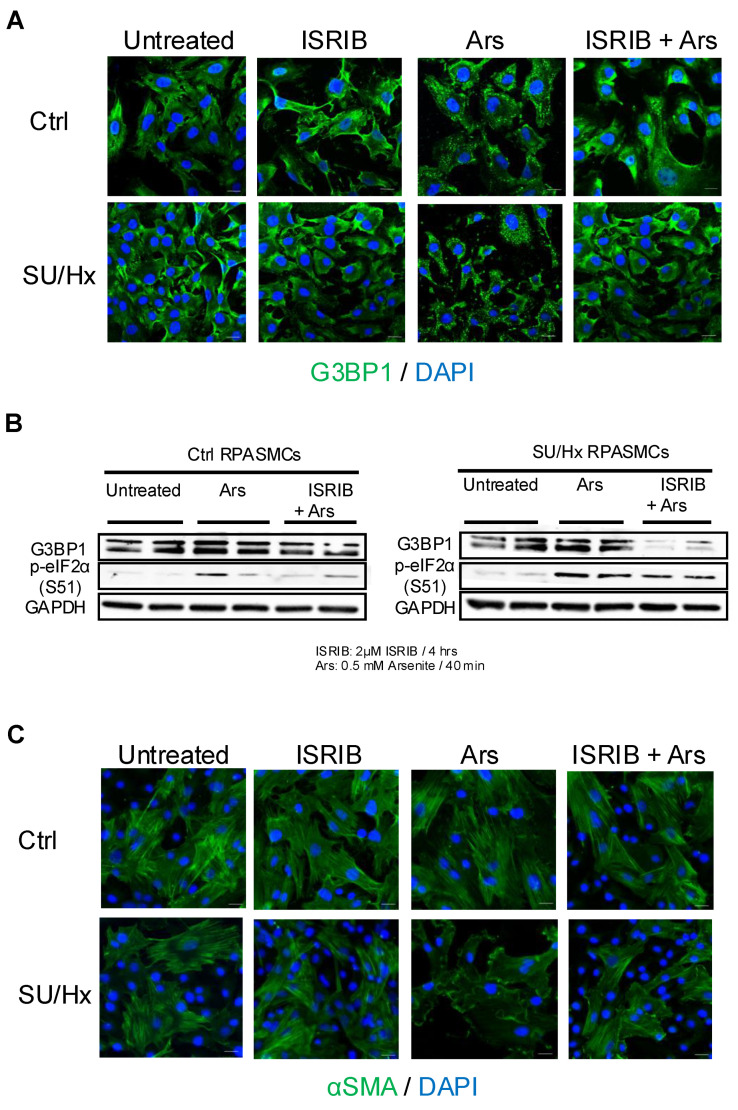
ISRIB treatment results in lower numbers of SGs after oxidative stress and restores the contractile phenotype of RPASMCs from SU/Hx animals. (**A**) RPASMCs from SU/Hx animals and controls were pre-treated with ISRIB for 4 h, then treated with 0.5 mM arsenite for 40 min and stained for G3BP1 to detect SGs. Nuclei were visualized with DAPI staining. Scale bar, 20 μm. (**B**) Immunoblots showing downregulation of G3BP1 and slight decrease in p-eIf2α when pre-treated with ISRIB for 4 h, then treated with 0.5 mM arsenite for 40 min in RPASMCs isolated from SU/Hx animals compared to control cells. (**C**) RPASMCs from SU/Hx animals and controls were pre-treated with ISRIB for 4 h, then treated with 0.5 mM arsenite for 40 min and stained for αSMA to detect stress fibers and focal adhesions. Nuclei were visualized with DAPI staining. Scale bar, 20 μm.

We successfully inhibited SG formation by arsenite in healthy and diseased RPASMCs with ISRIB treatment that blocks the translational effects of eIF2α phosphorylation [24,25], as assessed by G3BP1 and p-eIF2a abundance in Figure 5A,B. While G3BP1 abundance was more prominently decreased in response to ISRIB in diseased cells compared to controls, p-eIF2α levels were only partially decreased after ISRIB treatment in PASMCs from SU/Hx animals, compared to the complete decrease in PASMCs from control animals (Figure 5B). Treatment of SU/Hx RPASMCs with ISRIB also decreased arsenite-induced actin reorganization, as shown by decreased focal adhesions in Figure 5C.

### 3.6. G3BP1 Downregulation Increases Apoptosis and Inhibits Proliferation of PASMCs from Rats with SU/Hx-Induced PH

**Figure 6:**
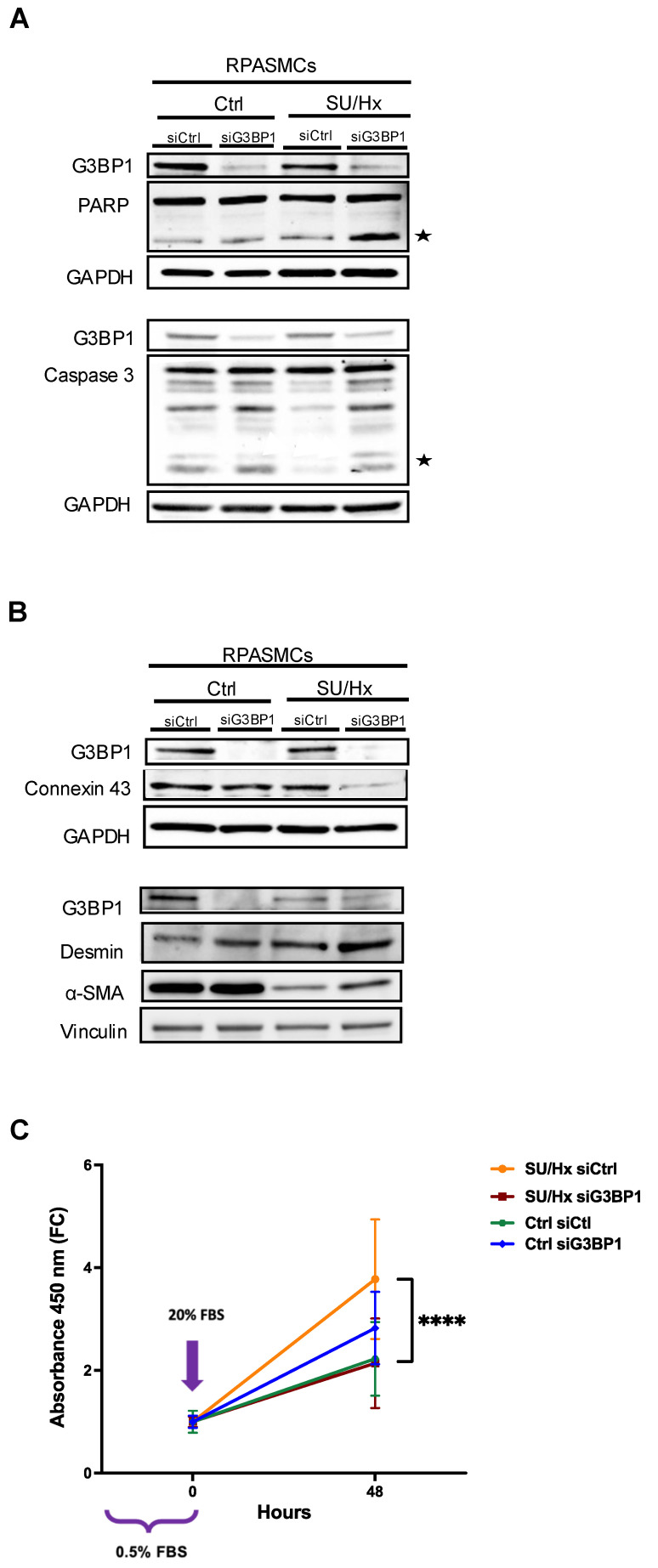
G3BP1 downregulation increases apoptosis, restores a contractile phenotype, and decreases proliferation of RPASMCs isolated from SU/Hx animals compared to control animals. (**A**) Immunoblots confirming downregulation of G3BP1 in RPASMCs isolated from SU/Hx animals and control cells. Knockdown of G3BP1 induces apoptosis (as assessed by cleaved PARP and cleaved caspase 3, indicated by stars) and, respectively, inhibits synthetic markers (Connexin 43) and increases contractile marker (desmin and α-SMA) expression in RPASMCs isolated from SU/Hx animals compared to control cells (**B**). (**C**) Knockdown of G3BP1 decreased the number of RPASMCs isolated from SU/Hx animals compared to control cells at 48 h (Cell proliferation reagent WST-1); **** *p* < 0.0001.

We used siRNA for genetic ablation of G3BP1 (Figure 6A). To determine whether G3BP1 affects apoptosis in RPASMCS from SU/Hx animals, we examined cleaved caspase 3—because accumulation of caspase 3 in SGs inhibits apoptosis [26]—and cleaved PARP by immunoblot, and found that G3BP1 knockdown induced an increase in cleaved caspase 3 and cleaved PARP in RPASMCs from SU/Hx animals, but not in PASMCs from control animals (Figure 6A). 

We also found that knocking down G3BP1 in RPASMCs from SU/Hx animals led to decreased abundance of the synthetic marker Connexin 43 [27] and increased abundance of the contractile markers desmin and α-SMA (alpha smooth muscle actin) (Figure 6B). As shown in Figure 6C, G3BP1 knockdown also selectively inhibited the proliferation of PASMCS isolated from SU/Hx animals, whereas it increased proliferation in PASMCs from control animals.

### 3.7. SG Protein Puncta in Lungs of Patients with PAH, Increased SGs in Human PAH-PASMCs, and Decreased Proliferation Upon Genetic Ablation of G3BP1

**Figure 7:**
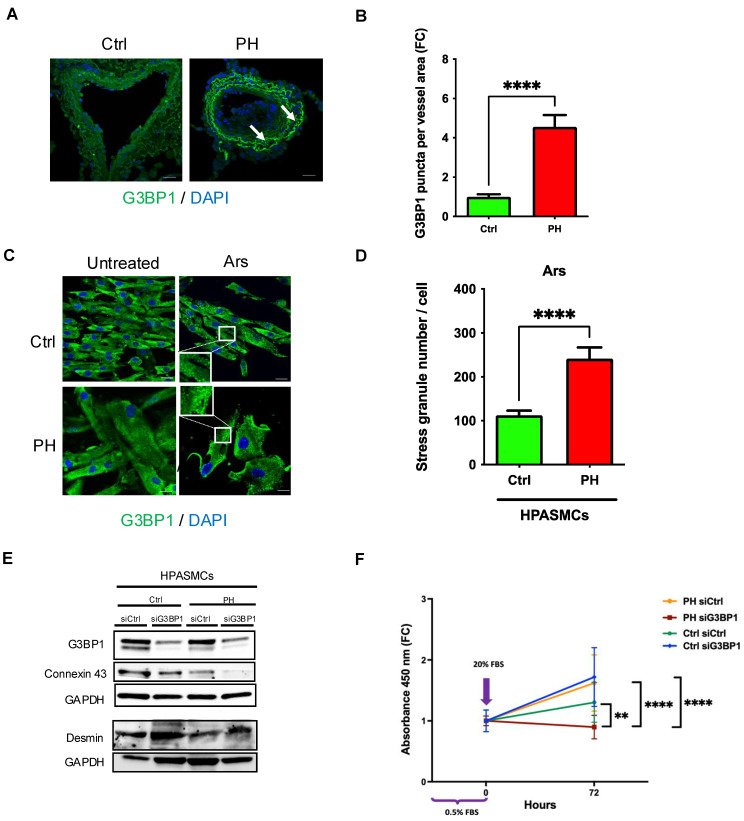
SG puncta in pulmonary blood vessels in lungs from patients with PAH compared to controls; PASMCs from the same patients have more SGs after acute oxidative stress and G3BP1 downregulation decreases their proliferation compared to controls. (**A**) Paraffin-embedded sections of lungs from patients with PAH and healthy donors stained with G3BP1. Arrows indicate G3BP1 puncta, quantified with CellProfiler (**B**). (**C**) PASMCs from patients with PAH and healthy donors were treated with 0.5 mM arsenite for 40 min and stained for G3BP1 to detect SGs, quantified with CellProfiler (**D**). Nuclei were visualized with DAPI staining. Scale bar, 20 μm; **** *p* < 0.0001 (*n* = 45 cells/cell type). (**E**) Immunoblots confirming downregulation of G3BP1 in hPASMCs isolated from patients with PAH and healthy donors. Knockdown of G3BP1 inhibits synthetic marker (Connexin 43) and increases contractile marker (desmin) expression in PASMCs isolated from patients with PAH and healthy donors. (**F**) Knockdown of G3BP1 decreased the number of HPASMCs isolated from patients with PAH compared to healthy donors at 72 h (Cell proliferation reagent WST-1); ** *p* < 0.01, **** *p* < 0.0001.

Paraffin-embedded lung tissues from patients with PAH and healthy donors were stained for G3BP1 and showed formation of G3BP1 puncta in pulmonary blood vessels in patient samples compared to controls (Figure 7A,B) (Supplementary Table S1). There was a >2-fold increase in SGs in response to acute oxidative stress with arsenite treatment in human PAH-PASMCs compared to control cells (Figure 7C,D).

Similarly, for PASMCs from SU/Hx animals, we found that genetic inhibition of G3BP1 (using siRNA) (Figure 7E) selectively inhibits the proliferation of PASMCs isolated from PAH patients compared to PASMCs from healthy donors (Figure 7F). We also found that G3BP1 knockdown in PAH-PASMCs resulted in decreased abundance of the synthetic marker Connexin 43 and increased abundance of the contractile marker desmin (Figure 7E) compared to PASMCs from healthy controls.

## 4. Discussion

We present novel findings on the potential role of SG formation in PAH pathogenesis in a preclinical model and in human PAH tissues and cells. We report for the first time a link between SG formation and abnormal vascular phenotypes in animal and human pulmonary vascular smooth muscle cells and increased abundance of SG puncta in right ventricles and skeletal muscles in the preclinical model of severe PH induced by SU/Hx in adult rats. Furthermore, we show that ACTZ treatment, an intervention known to restore a contractile pulmonary vascular smooth muscle cell phenotype and ameliorate experimental PAH, is associated with the inhibition of SG formation. Although preliminary trials of ACTZ in human PAH have not shown a sustained benefit in functional outcomes [28,29], whether ACTZ restores a contractile PASMC phenotype and improves responsiveness to pulmonary vasodilators in human PAH has not been evaluated. 

Stress granules are a subset of ribonucleoprotein granules that are normally a dynamic cellular compartment that contributes to cellular homeostasis by delaying the translation of specific transcripts under conditions of cellular stress. Defects in granulostasis lead to the accumulation of solid, aggregate-like inclusions and cellular dysfunction and have been associated with age-related neurodegenerative disorders, cancer, auto-immune disorders, and the response to viral infections [30,31,32,33,34]. However, the role of SGs in vascular homeostasis and specifically in PAH are only beginning to be examined. 

Our main findings pertain to the association between pulmonary vascular smooth muscle cell homeostasis and SG formation. Several lines of evidence support that a dedifferentiated, proliferative, migratory and apoptosis-resistant phenotype is characteristic of pulmonary hypertension in both preclinical models and human PAH [2,3]. Here, we demonstrate that this abnormal phenotype is associated with increased abundance of SGs and, more importantly, using a pharmacologic and a genetic approach, we demonstrate a causal relationship between SG formation and abnormal PASMC homeostasis. We used ISRIB (integrated stress response inhibitor) as an inhibitor of SG-dependent pathways and found that it inhibited arsenite-induced actin reorganization, as shown by decreased focal adhesions, similar to focal adhesion kinase (FAK) inhibition in PAH-PASMCs [35,36]. 

Our findings are in agreement with Wang et al., who demonstrated targeting the integrated stress response using a different pharmacologic approach, blocked vascular smooth muscle cell dedifferentiation and proliferation, and mitigated both restenosis and thrombosis in animal models [37]. Another study by Onat et al. showed that ISRIB treatment alleviated atherosclerosis in mice [38]. Given that ISRIB’s effects may be non-specific, we also used a siRNA approach to block G3BP1, a key component of SG assembly. In agreement with our results using the pharmacologic inhibitor, we found that G3BP1 knockdown resulted in a more contractile and less synthetic phenotype, decreased proliferation, and increased apoptosis in both rat and human PASMCs. A study by Herman et al. showed that SGs accumulate in vascular smooth muscle cells and macrophages during atherosclerosis in mouse models. In addition, this study showed that reductions in G3BP1 by siRNA significantly altered expression profiles of apoptotic, inflammatory, and proliferative genes in human vascular smooth muscle cells [39]. Taken together, these results are in agreement with our results and indicate that SG formation is a common feature of diseases of vascular homeostasis. 

In several of our in vitro studies, we used arsenite exposure as an acute oxidative cellular stressor. This is pertinent to pulmonary hypertension since oxidative stress was previously shown to be associated with pulmonary vasculature and RV remodeling [40]. We previously showed that treatment with the carbonic anhydrase inhibitor ACTZ ameliorates the hemodynamic components and RV metabolic dysfunction in experimental PAH [3,17] and restores a contractile PASMCs phenotype in vivo [3]. Another study by Prouillac et al. [41] suggests that ACTZ may have reactive oxygen species (ROS)-scavenging properties. In agreement with these prior studies, we now show that ACTZ inhibits SG component assembly in tissues from SU/Hx animals in vivo and in PASMCs after oxidative stress in vitro, suggesting that ACTZ also acts as an antioxidant or a SG-modulating compound. This is corroborated by a study by Shimoda et al. who reported that ACTZ prevents hypoxia-induced changes in PASMCs by reducing reactive oxygen species production and the subsequent triggering of Ca2+ release from sarcoplasmic reticulum [42]. 

Beyond the effects of SGs in vascular homeostasis, in the present study we describe increased RNP granulopathy in right ventricles in SU/Hx animals compared to controls and ACTZ-treated SU/Hx animals. The significance of this observation remains to be determined since the role of SGs in cardiomyocyte homeostasis is incompletely understood. A transcriptomic study in primary neonatal cardiomyocytes supports that G3BP1 induces hypertrophic transcriptome and regulates cardiomyocyte hypertrophy [43]. A study by Schneider et al. reported an association between RNP granule pathobiology and heart failure in gene-edited pigs and patients with dilated cardiomyopathy caused by RBM20 mutation [44]. Another study by Dong et al. showed a cytoprotective role for G3BP1 in atrial fibrillation [45]. 

Finally, we describe increased RNP granulopathy in soleus muscles in SU/Hx animals compared to controls and ACTZ-treated SU/Hx animals. We previously reported decreased endurance in this model that correlated with the formation of sarcomeric aggregates in soleus muscles [20], so it is possible that the formation of SG puncta in the same tissues is related to skeletal muscle dysfunction, but additional studies are needed to explore this hypothesis. The role of SGs in skeletal muscle homeostasis has been explored in the setting of congenital myopathies such as Welander distal myopathy (WDM) [46,47,48] and Matrin 3 myopathy [49]. In both of these disorders, there is detection of abnormal SG dynamics that may be related to disease pathogenesis. 

In summary, this work identifies a potential novel pathogenetic mechanism and therapeutic target in PAH research by the identification of abnormal SG homeostasis in pulmonary vascular smooth muscle cells, right ventricles, and skeletal muscles in a clinically relevant preclinical model of PAH, and in pertinent human PAH tissues. Further studies are needed to delineate how stress granule biology contributes to cellular mechanisms of disease and how this knowledge can be leveraged therapeutically.

## 5. Conclusions

The novel concept that is put forward here is that stress granules may contribute to the pathogenesis and progression of PAH in settings of cellular stress, as depicted in the graphical abstract (created with smart.servier.com).

## Data Availability

The data presented in this study are available in the article or Supplementary Materials.

## Supplementary Materials

The following supporting information can be downloaded at: https://www.mdpi.com/article/10.3390/cells13211796/s1. Figure S1: Experimental timeline. Figure S2: Hemodynamic characteristics of experimental pulmonary hypertension. Table S1: Demographics and clinical information of human patients with IPAH and controls.

## Abbreviations

ACTZ	Acetazolamide
Ars	Arsenite (NaAsO_2_)
α-SMA	Alpha smooth muscle actin
BW	Body weight
Caprin1	Cycle associated protein 1
DAPI	4′,6-diamidino-2-phenylindole
DMSO	Dimethyl sulfoxide
G3BP1	Ras-GAP SH3-domain-binding protein 1
GAPDH	Glyceraldehyde-3-Phosphate Dehydrogenase
FAK	Focal adhesion kinase
FBS	Fetal bovine serum
FI	Fulton’s Index
HPASMCs	Human pulmonary artery smooth muscle cells
IPAH	Idiopathic pulmonary arterial hypertension
ISRIB	Integrated stress response inhibitor
LVSP	Left ventricular systolic pressure
PAH	Pulmonary arterial hypertension
PARP	Nuclear poly (ADP-ribose) polymerase
PASMCs	Pulmonary artery smooth muscle cells
p-eIF2α	Phospho-eukaryotic translation initiation factor 2A
PHBI	Pulmonary Hypertension Breakthrough Initiative
RBPs	RNA-binding proteins
ROS	Reactive oxygen species
RPASMCs	Rat pulmonary artery smooth muscle cells
RV	Right ventricular weight
RVH	Right ventricular hypertrophy
RVSP	Right ventricular systolic pressure
SG	Stress granule
siRNA	Small interfering RNA
SU/Hx	Sugen/Hypoxia
TBST	Tris-buffered saline with Tween-20
WDM	Welander distal myopathy
