# Supplementary material for: Stress Granule Assembly in Pulmonary Arterial Hypertension
Source: Cells. 2024 Oct 30;13(21):1796. doi: 10.3390/cells13211796 (PMC11544768; doi:10.3390/cells13211796)
Supplement: Supplementary file 1 [file cells-13-01796-s001.zip › cells-3233853-supplementary.pdf]

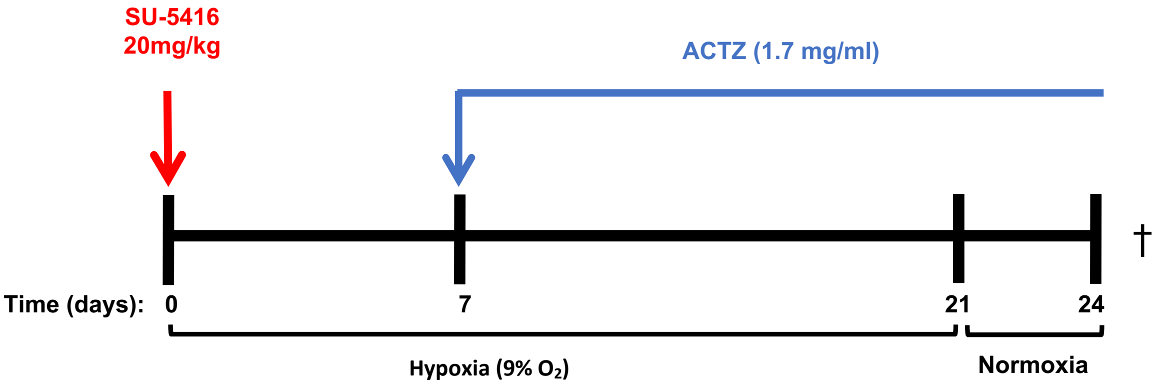

Supplementary figure S1.

Supplementary figure S1. Experimental timeline. Rats were administered an injection with Sugen 5416 (SU; 20 mg/kg s.c.) and housed under hypoxic condition (9% O_2) for 3 weeks followed by 3 days under normoxic condition (21% O_2). Treatment was administered with ACTZ (1.7 mg/ml) *ad libitum* in the drinking water from Day 7 to Day 24. Control rats were treated with ACTZ in normoxia for the same time period. † This represents the study endpoint when animals were killed.

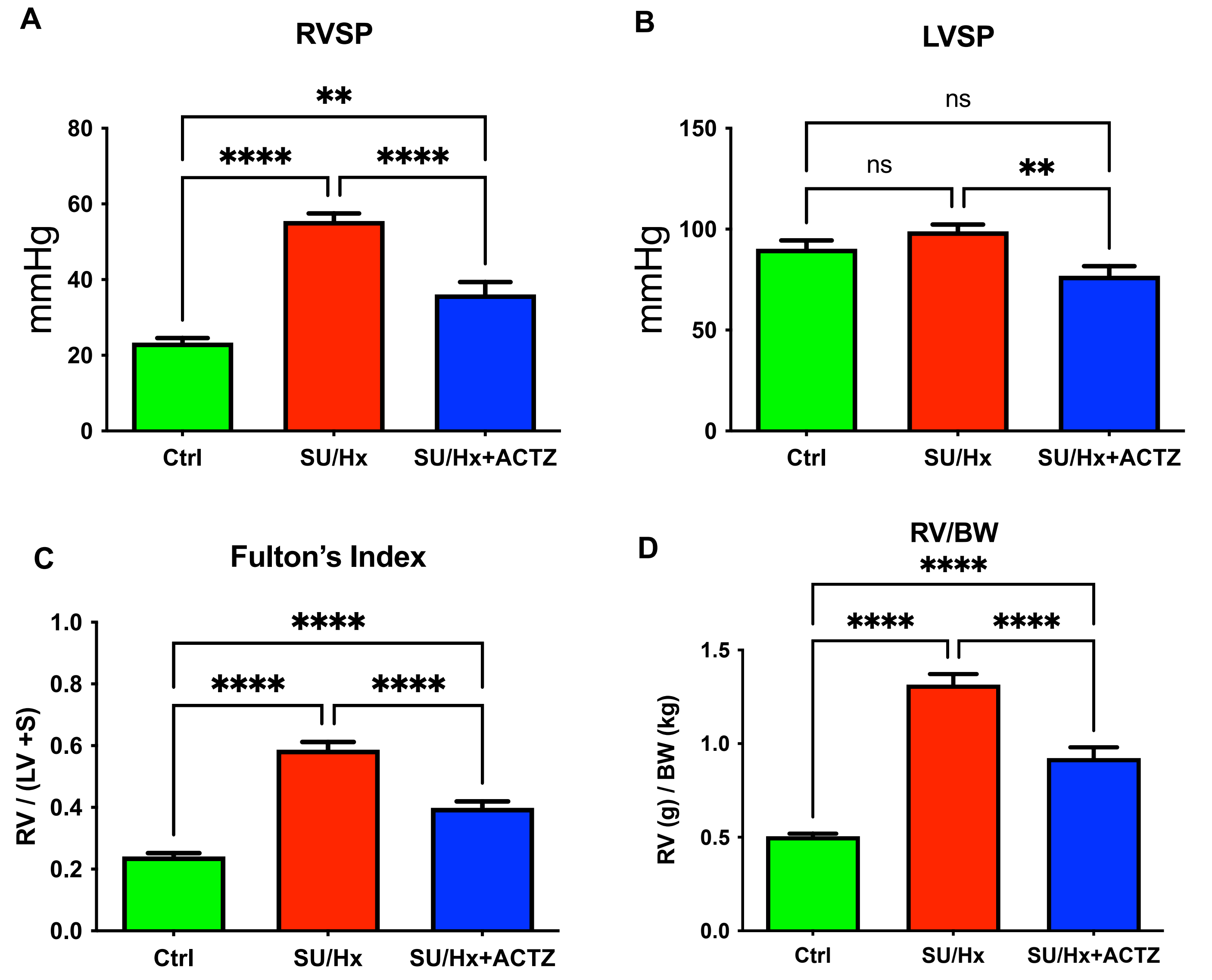

Supplementary figure S2.

Supplementary figure S2. Hemodynamic characteristics of experimental pulmonary hypertension. **(A)** Right Ventricular Systolic Pressure (RVSP), **(B)** Left Ventricular Systolic Pressure (LVSP), **(C)** Right ventricular hypertrophy assessed by Fulton's Index (ratio of right ventricular weight to left ventricular and septal weight) and **(D)** ratio of right ventricular weight to total body weight (RV/BW). n = 9 -15 animals per group. Statistical analysis by one- way ANOVA. Values are expressed as the mean ± SEM (***p*<0.01 and *****p*<0.0001).

CONTROL					
TP Number	Age	Race	Sex	Procedure	Category
12-09-P039e	56YR	White	Female	Surgery	Control - lung w/ M-L arteries
13-01-P049a	51YR	White	Male	Surgery	Control - lung w/ M-L arteries
IPAH					
11-06-P061d	56YR	White	Female	Transplant	IPAH - lung w/ M-L arteries
12-06-P009e	51YR	White	Male	Transplant	IPAH - lung w/ M-L arteries

Supplementary Table S1.

Supplementary Table S1. Demographics and clinical information of human patients with IPAH and controls.
